# Assessing the Protective Dose of a Candidate DIVA Vaccine against Classical Swine Fever

**DOI:** 10.3390/vaccines9050483

**Published:** 2021-05-10

**Authors:** Tinka Jelsma, Jacob Post, Erwin van den Born, Ruud Segers, Jeroen Kortekaas

**Affiliations:** 1 Wageningen Bioveterinary Research, 8221 RA Lelystad, The Netherlands; tinka.jelsma@wur.nl (T.J.); jacob.post@wur.nl (J.P.); 2 MSD Animal Health, 5830 AA Boxmeer, The Netherlands; erwin.van.den.born@merck.com (E.v.d.B.); ruud.segers@merck.com (R.S.); 3 Laboratory of Virology, Wageningen University, 6708 PB Wageningen, The Netherlands

**Keywords:** classical swine fever virus, pestivirus, swine, vaccine, DIVA

## Abstract

Classical swine fever is a highly contagious and deadly disease in swine. The disease can be controlled effectively by vaccination with an attenuated virus known as the “Chinese” (C)-strain. A single vaccination with the C-strain provides complete protection against highly virulent isolates within days after vaccination, making it one of the most efficacious veterinary vaccines ever developed. A disadvantage of the C-strain is that vaccinated animals cannot be serologically differentiated from animals that are infected with wild-type Classical swine fever virus. Previously, a C-strain-based vaccine with a stable deletion in the E2 structural glycoprotein was developed, which allows for differentiation between infected and vaccinated animals (DIVA). The resulting vaccine, which we named C-DIVA, is compatible with a commercial E2 ELISA, modified to render it suitable as a DIVA test. In the present work, three groups of eight piglets were vaccinated with escalating doses of the C-DIVA vaccine and challenged two weeks after vaccination. One group of four unvaccinated piglets served as controls. Piglets were monitored for clinical signs until three weeks after challenge and blood samples were collected to monitor viremia, leukocyte and thrombocyte levels, and antibody responses. The presence of challenge virus RNA in oropharyngeal swabs was investigated to first gain insight into the potential of C-DIVA to prevent shedding. The results demonstrate that a single vaccination with 70 infectious virus particles of C-DIVA protects pigs from the highly virulent Brescia strain.

## 1. Introduction

The genus Pestivirus of the family *Flaviviridae* includes classical swine fever virus (CSFV), border disease virus (BDV) and bovine viral diarrhoea virus (BVDV). Pestiviruses that belong to the latter two species are found in both swine and ruminants, whereas CSFV infects exclusively domesticated pigs, feral pigs and wild boar (*Sus scrofa*) [1]. CSFV typically enters the host via ingestion, or less commonly via the conjunctiva. Entry via the oronasal route is followed by infection of epithelial cells of the tonsillar surface. After primary replication in these cells, the virus targets macrophages and dendritic cells. Infection may remain subclinical or run an acute, subacute, late-onset, or chronic course. The acute form of the disease manifests with fever, loss of appetite, dullness, unsteady gait and constipation followed by diarrhoea. Several days after the onset of fever, cyanotic discolorations may be observed on the ears, abdomen and inner thighs. These manifestations are caused by thrombosis and endothelial damage, which may also result in hemorrhagic diatheses and petechiation. Gross pathological findings include petechial to ecchymotic hemorrhages on lymph nodes and kidneys and also on the larynx, epiglottis, heart, urinary bladder, intestinal mucosa, serous and mucous membranes and skin [2,3]. Transplacental transmission may result in congenital malformations, the birth of weak piglets, stillbirth, or abortion. Importantly, infections during the second trimester of gestation may result in persistently infected piglets, which may shed virus to the environment until succumbing to a late-onset form of the disease [4,5].

CSF is endemic to parts of Europe, Central and South America, Asia and Africa, whereas North America, Australia and New Zealand are free from the disease [3]. Due to the risk of large transboundary outbreaks with considerable economic damage, the World Organisation for Animal Health (Office International des Epizooties (OIE)) has to be notified when CSF is diagnosed in the field. Diagnosis of CSFV infection can be performed by PCR on organ or blood samples during the acute phase of the infection, or via the detection of antibodies by ELISA or virus neutralization assay. Commercially available CSF ELISAs detect antibodies against either the E2 or E^RNS^ structural glycoprotein [6]. Although antibodies against E^RNS^ may appear a few days before E2 antibodies, E2 ELISAs are of much higher sensitivity after convalescence. Importantly, E^RNS^ ELISAs do not differentiate between CSFV and related pestiviruses, explaining that these ELISAs are recommended for use only at herd level [7].

Epizootics of CSF can be controlled effectively by vaccination with live-attenuated vaccines, of which the lapinized “Chinese” or “C” strain is most widely used. A single vaccination with a C-strain-based vaccine can provide lifelong immunity with an onset as early as 2–3 days after vaccination [1]. In the 1980s, vaccination with C-strain played an important role in the eradication of CSF from Europe and its use across the world has contributed to its reputation as one of the most effective and safe veterinary vaccines ever developed. The only disadvantage of the C-strain vaccine is the inability to differentiate infected from vaccinated animals (DIVA) by ELISA. Therefore, vaccination with C-strain may result in trade restrictions with significant economic consequences.

With the aim of developing a vaccine that enables DIVA, a subunit vaccine was developed in the late 1990s. This vaccine is based on the E2 glycoprotein, which is the dominant target of protective, neutralizing antibodies [8]. Although the E2 vaccine is highly efficacious after a single vaccination, the C-strain vaccine is superior with respect to both onset and duration of immunity [1]. To develop a DIVA vaccine with comparable efficacy to the C-strain, several live-attenuated vaccines were developed in recent decades [9]. These vaccines can be roughly divided into those that are compatible with E2 ELISAs and those that are compatible with E^RNS^ ELISAs as DIVA assays. The most efficacious of these vaccines are chimeric pestiviruses in which the gene encoding either E2 or E^RNS^ is replaced for the corresponding gene of another pestivirus species [10,11,12]. One of these vaccines, named CP7_E2alf, is based on a BVDV strain carrying E2 of CSFV and was brought to market by Zoetis as Suvaxyn CSF Marker [13]. Suvaxyn CSF Marker is a highly efficacious and safe vaccine. However, a shortcoming of this vaccine is that its applicability as a DIVA vaccine depends on E^RNS^ ELISAs which, due to its limited specificity and sensitivity, should only be used at a herd level [7,14]. We previously reported the creation of a C-strain vaccine virus that contains a stable deletion in the E2 ectodomain [15]. We subsequently demonstrated that a single vaccination with the resulting vaccine virus, previously named vFlc-ΔPTa1 and now referred to as C-DIVA, provides protection against the highly virulent Brescia strain and strongly reduces or even prevents shedding of the wild-type virus [16]. While this former study demonstrated that the antibody response induced by C-DIVA could not be differentiated from that induced by wild-type CSFV with a commercial E2 ELISA, a novel DIVA ELISA was developed that was shown to allow this differentiation [17]. In the present work, we determined the 50% protective dose (PD50) of the C-DIVA vaccine and found that even a very low dose of 70 infectious particles protects pigs from mortality, clinical signs and viremia when challenged with the highly virulent Brescia strain. The results suggest that the C-DIVA vaccine holds great promise as a cost-effective, next-generation CSF vaccine.

## 2. Materials and Methods

### 2.1. Housing, Vaccination and Challenge

The animal study was approved by the Wageningen Bioveterinary Research (WBVR) ethical committee (permit #2014199) and humane endpoints were defined. A total of 36 piglets, approximately 8 weeks of age at the start of the experiment, were randomly allotted to 4 treatment groups of 8 animals and one control group of 4 animals. Piglets were purchased from a high-health farm and were not vaccinated. The sows on this farm were vaccinated before farrowing against *E. coli* and *Clostridium*, rotavirus, and streptococcus. One week after farrowing, sows were vaccinated against porcine parvovirus and swine erysipelas. Each group of piglets was housed in an isolation unit of 9 m^2^ in the High Containment facility of WBVR. Pigs were fed once per day and potable water was available ad libitum.

After one week of acclimatization, treatment groups 1–4 were vaccinated once with a dose of 1, 2, 3 or 4 log_10_ 50% Tissue Culture Infective Dose (TCID_50_), respectively. Inoculation was performed via the intramuscular route, by injecting 1 mL culture medium containing the vaccine virus, into the left side of the neck. Vaccine virus titers were determined on SK6-T7 cells [18]. The control group was mock-vaccinated via the same inoculation route, by injection of 1 mL PBS. After vaccination, all pigs were observed daily for clinical signs and occurrence of local reactions, and rectal temperatures were recorded. EDTA blood and serum samples were collected on days 0 (prior to vaccination), 8, 14 (prior to challenge), 21, 25, 28, 32 and 36. On days 16, 18, 23 and 30, only EDTA blood samples were collected. Two weeks after vaccination, all animals were challenged via the intranasal route with 3.84 log_10_ TCID_50_ of the highly virulent CSFV strain Brescia 456610 [19], by applying 0.5 mL in each nostril, representing approximately 100 times the 50% lethal dose (LD50). Samples of the challenge virus before and after administration were titrated to confirm the challenge dose.

During the following observation period of 24 days, all animals were observed daily for clinical signs and rectal temperatures were recorded. The severity of clinical signs was scored using a previously defined list of 10 CSF-specific criteria [20]. Serum samples were taken at regular timepoints for serology (E2 and E^RNS^ ELISA, and neutralisation-peroxidase-linked-assay (NPLA)) and EDTA blood was collected for isolation of white blood cells (WBC) for virus isolation. EDTA blood samples were also used to monitor leukocyte and thrombocyte levels. Oropharyngeal fluid (OPF) samples were taken on days 16, 18, 21, 23, 25, 28, 30, 32 and 36. At the end of the observation period (22 days after challenge), all surviving animals were euthanized by electric stunning, immediately followed by exsanguination. Post-mortem examination was performed on all animals that were euthanized or that succumbed during the experiment.

### 2.2. Laboratory Analyses

#### 2.2.1. Serology

To ensure that pigs were pestivirus seronegative before the start of the study, serum samples taken from all animals were tested for antibodies against pestiviruses 13 days before vaccination with a “pan-pesti” ELISA (NS3 BVDV ELISA, Prionics, Lelystad, the Netherlands), according to the instructions of the manufacturer. During the study, all serum samples were analysed with the CSFV E2 ELISA (IDEXX, Hoofddorp, the Netherlands) according to the manufacturers’ instructions and by the DIVA ELISA as described [17]. Briefly, 50 µL of serum was added to a 25 µL 2x concentrated dilution buffer (kindly provided by IDEXX) and 25 µL baculovirus-produced purified ectodomain of the C-DIVA E2 protein (100 μg/mL), and incubated for 2 h at RT. The rest of the procedure was performed according to the protocol of the CSFV E2 ELISA. To determine the presence of CSFV-neutralising antibodies, serum samples were tested by NPLA as described [21].

#### 2.2.2. Quantitative Real-Time PCR

Leukocytes were isolated and suspended in tissue culture medium supplemented with 5% Fetal Bovine Serum (FBS) and antibiotics. The leukocytes were stored as aliquots of 1 mL at −20 °C until RT-qPCR analysis. Viral RNA from leukocytes and OPF was extracted using the MagNA Pure1 system and the MagNA Pure1 LC kit (Roche). The isolated RNA was used for quantitative reverse transcriptase PCR (RT-qPCR) using a LightCycler and primers specific for the 5′-nontranslated region of the CSFV genome [22]. A standard curve was developed using the CSFV strain Brescia of known titre determined on SK6 cells [23].

#### 2.2.3. WBCs and Thrombocyte Quantification

White blood cells (WBCs) and thrombocytes were counted from the EDTA blood samples using the Coulter counter (Medonic 570). Leukopenia and thrombocytopenia were defined as WBC and thrombocyte levels below 10 × 10^9^ cells/L and 200 × 10^9^ cells/L, respectively.

#### 2.2.4. Statistical Analyses

Statistical analyses were performed using GraphPad Prism 5. Two-way ANOVA including Bonferroni’s Multiple Comparison Test was applied to evaluate significant differences between vaccinated groups and the mock-vaccinated group. *p* values < 0.05 are considered significant.

#### 2.2.5. PD50 Validity and Determination

The PD50 study was found valid as all mock-vaccinated animals developed clinical signs typical of CSFV within 21 days. The 50% protective dose (PD50) was determined for the following three parameters: mortality, clinical signs (including body temperature) typical for CSF and viremia and calculated according to the method described by Spearman and Kärber [24,25].

## 3. Results

### 3.1. Body Temperatures

**Figure 1:**
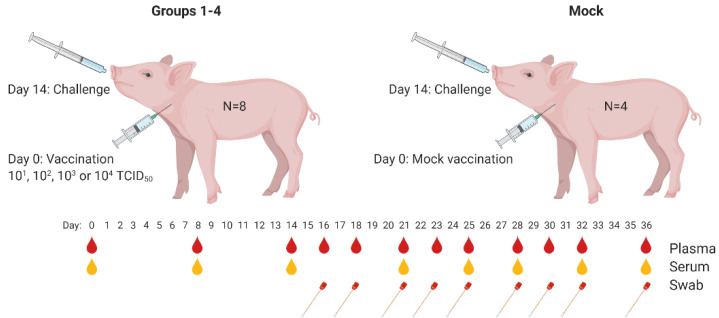
Experimental outline and procedures. Plasma samples (red droplets) were used for detection of viral RNA and serum (yellow droplets) for detection of antibodies by ELISA and neutralization assay. Swabs were used for detection of viral RNA by RT-qPCR. Cartoon created with BioRender.com.

**Figure 2:**
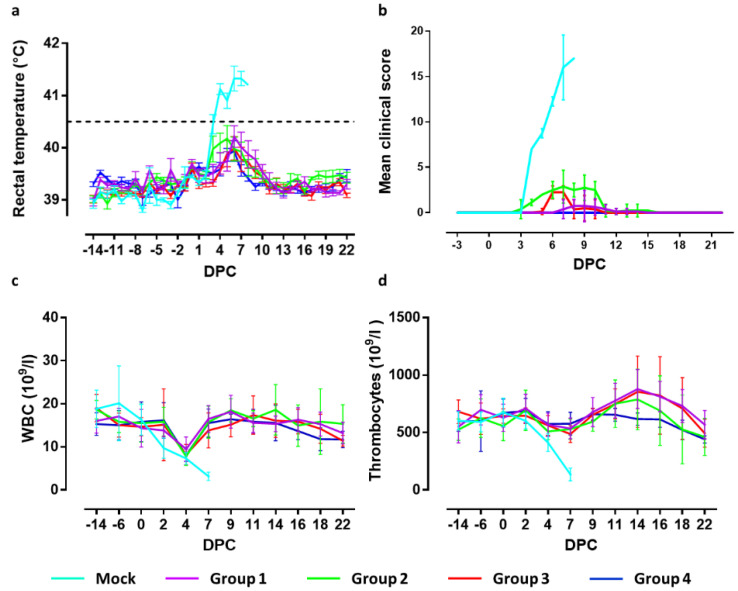
Clinical signs and blood cell counts of mock-vaccinated and C-DIVA-vaccinated animals following challenge infection with the highly virulent CSFV strain Brescia. (**a**) Mean rectal temperatures. Temperature above the dashed line is defined as fever. (**b**) Mean cumulative clinical scores. (**c**) Mean WBC counts. (**d**) Mean thrombocyte counts. Error bars represent SD. DPC; days post challenge.

To determine the 50% protective dose (PD50) of the C-DIVA vaccine, groups of eight animals were vaccinated once via the intramuscular route with a dose of 10^1^, 10^2^, 10^3^, or 10^4^ TCID_50_ of C-DIVA, designated groups 1, 2, 3, and 4, respectively. The control group (Mock) of four animals was mock-vaccinated (Figure 1). Fever, defined as a rectal temperature above 40.5 °C, was first noted in two mock-vaccinated animals 3 days post challenge infection (3 DPC). On 4 DPC, all mock-vaccinated animals had fever (40.8–41.9 °C) which persisted until the animals were euthanized (Figure 2a). In group 4, one animal developed a body temperature of 41.2 °C for one day (6 DPC). All other animals in this group showed normal body temperatures after challenge until the end of the experiment. In group 3, two animals maintained normal body temperatures throughout the experiment. The other animals all developed body temperatures between 40.7 and 40.9 °C for one day between 5 and 7 DPC. In group 2, two animals had normal body temperatures until the end of the in-life phase. The other animals from this group developed body temperatures between 40.7 and 40.9 °C for two or three days in the period 3–8 DPC. In group 1, four animals maintained normal body temperatures throughout the study. One animal developed a body temperature of 41 °C on day 3 prior challenge and of 40.5 °C on 6 DPC. Two animals developed temperatures of 40.7–41.2 °C on two successive days between 6–8 DPC. One animal developed temperatures of 40.5–41.3 °C on 7 successive days from 3–9 DPC. Differences in body temperatures between the mock-vaccinated group and groups 4, 3 and 1 were significant (*p* < 0.001) at 3–7 DPC. Significant differences between the mock-vaccinated group and group 2 were found on 4 (*p* < 0.001), 5 (*p* < 0.01), 6 and 7 DPC (*p* < 0.001). Individual body temperatures are presented in Figure S1 and Table S1.

### 3.2. Clinical Signs

Clinical signs manifested as increased respiration, decreased activity, body tension, anorexia, general weakness, constipation, redness of the skin and reddened eyes. Three animals of the mock-vaccinated group were euthanized at 7 DPC and the fourth on day 8, after reaching a humane end-point. The severity of clinical signs was scored using a previously defined list of 10 CSF-specific criteria [20]. The cumulative clinical scores are depicted in Figure 2b. Pigs of group 4 developed no clinical signs after challenge infection. In group 3, 7 animals showed a mild loss of appetite and slightly reduced activity at 6–7 DPC. One animal showed a minor loss of appetite one day earlier, which continued until 10 DPC. This animal also developed constipation between 9 and 10 DPC. From 11 DPC until the end of the in-life phase, no further clinical signs were observed in this group. Pigs of group 2 showed mild loss of appetite, starting at 3–4 DPC, and slightly reduced activity between 5 and 10 DPC. One animal developed constipation until 14 DPC. From 15 DPC until the end of the in-life phase, no clinical signs were observed in this group. Two animals of group 1 developed no clinical signs following challenge infection. Two other animals were slow on one day (8 DPC) and remained free of clinical signs until the end of the study. One animal displayed reduced activity, mild breathing problems and loss of appetite for one day (9 DPC). The remaining animals showed reduced activity and mild loss of appetite from 7–13 DPC. From 14 DPC until the end of the study period, no further clinical signs were observed in this group.

Summarizing the clinical observations, significant differences (*p* < 0.001) were observed between the mock-vaccinated group and all vaccinated groups at 4–7 DPC. Significant differences (*p* < 0.001) were observed between groups 3 and 4 on 6 and 7 DPC, and group 2 on 4–10 DPC. Clinical signs in challenged animals of groups 4 and 1 differed significantly (*p* < 0.05) on 8 and 9 DPC. Significant differences (*p* < 0.001) were found comparing group 3 with group 2 on 4, 5, 8, 9 and 10 DPC and with group 1 on 6 and 7 DPC. Clinical signs in groups 1 and 2 differed significantly on 4 DPC (<0.01) and 5–10 DPC (<0.001).

### 3.3. Blood Cell Counts

Average WBC counts are depicted in Figure 2c. In the mock-vaccinated group, all control animals displayed leukopenia from 4–8 DPC, with counts varying from 9.4 × 10^9^ to 6.7 × 10^9^ cells/L. Leukopenia was observed at 4 DPC in seven animals from group 4. Leukopenia was observed in two animals from this group on 18 DPC and one on 19 DPC. Additionally, in group 3, leukopenia was observed on 4 DPC in seven animals. One animal was leukopenic on 7 and 22 DPC. In group 2, three animals did not develop leukopenia, whereas three animals were leukopenic on 4 DPC only. Two animals were leukopenic on 4 DPC and on DPC 16 and/or 18. In group 1, five animals were leukopenic on DPC 4, of which two additionally on 0 and 22 DPC. One animal had leukopenia only on 18 DPC. Two animals did not develop leukopenia.

The mean values of the thrombocyte counts are depicted in Figure 2d. All mock-vaccinated animals displayed thrombocytopenia on 7 DPC, with counts varying from 184 × 10^9^ to 61 × 10^9^ cells/L. None of the vaccinated animals developed thrombocytopenia post challenge, except for one animal in group 2 on 18 DPC.

In summary, significant differences in WBC numbers between the mock-vaccinated group and groups 2 and 4 were found on 2 DPC (*p* < 0.05) and 7 DPC (*p* < 0.001), whereas significant differences between the mock-vaccinated group and Groups 1 and 3 were observed on 7 DPC (*p* < 0.001). Thrombocyte levels of the mock-vaccinated group differed significantly from all vaccinated groups on 7 DPC (*p* < 0.001) and, on 14 DPC, thrombocyte levels significantly differed between group 4 and groups 3 (*p* < 0.05) and 1 (*p* < 0.01).

### 3.4. Detection of Viral RNA in Oropharyngeal Swabs by RT-qPCR

**Figure 3:**
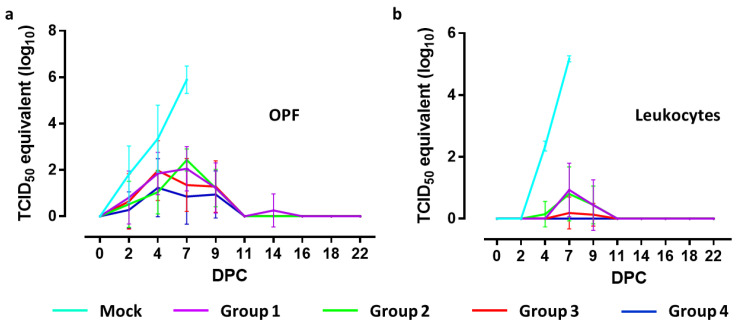
Detection and quantification of CSFV-specific RNA in OPF samples and leukocytes collected from vaccinated and mock-vaccinated pigs after challenge infection. Graphs depict means of viral RNA levels, as log_10_ TCID_50_/_mL_, detected in OPF samples (**a**) or isolated leukocytes (**b**). Error bars represent SD.

To quantify viral RNA levels in oropharyngeal swabs and leukocytes, a standard curve was created using known titers of CSFV strain Brescia determined by virus titration on SK6 cells. The results of the RT-qPCR are indicated as equivalent virus titers (TCID_50_-eq/mL) (Figure 3).

Two days after challenge, titers ranging from 2.16 to 2.69 log_10_ TCID_50_-eq/mL were detected in oropharyngeal fluid collected from three out of four mock-vaccinated animals, whereas one animal was still negative. During the following days, all piglets of this group were found positive, reaching maximum titers of 5.09 to 6.52 log_10_ TCID_50_-eq/mL on 7 DPC. All piglets in group 4 were positive at least on one day between 2 and 9 DPC. Five animals were positive on one day, two animals on two days and one animal on four successive days with titers ranging from 0.31 to 2.75 log_10_ TCID_50_-eq/mL. In group 3, three animals were positive on one day, one animal on two days, three animals on three days and one on four successive days, with titers ranging from 1.43 to 3.53 log_10_ TCID_50_-eq/mL. Piglets from group 2 were positive for at least one day between 2 and 9 DPC. One animal was positive only on 7 DPC, two animals on two days, four animals on three days and one animal on four days, with titers ranging from 1.15 to 3.21 log_10_ TCID_50_-eq/mL. In group 1, one animal remained negative throughout the in-life phase. One animal was positive on two days, three on three days and three animals on four days, with titers ranging from 1.52 to 3.05 log_10_ TCID_50_-eq/mL.

Comparing the mock-vaccinated group with group 4 revealed significant differences between titers from samples collected on 2 DPC (<0.05), 4 and 7 DPC (*p* < 0.001). Significant differences with group 3 were found at 7 DPC (*p* < 0.001), with group 2 at 4 and 7 DPC (*p* < 0.001) and with group 1 at 4 (*p* < 0.05) and 7 DPC (*p* < 0.01). No significant differences were found when titers from samples collected from groups 3 and 4 were compared. Significant differences between group 4 and groups 1 and 2 were found on 7 DPC (*p* < 0.01 and *p* < 0.001, respectively).

### 3.5. Detection of Viral RNA in Leukocytes by RT-qPCR

Four days after challenge, all mock-vaccinated animals contained viral RNA in isolated leukocytes, with titers ranging from 2.14 and 2.49 log_10_ TCID_50_-eq/mL. On 7 DPC, maximum titers ranging from 5.05 and 5.28 TCID_50_-eq/mL were detected.

Vaccinated pigs from group 4 remained negative for viral RNA in leukocytes during the entire experimental period. One pig from group 3 showed very low titers (max 1.48 log_10_ TCID_50_-eq/mL) on DPC 7 and 9, while the other animals in this group remained negative. In group 2, three animals remained negative, two animals were positive on one day and three animals on two days in the period 4–9 DPC, with titers ranging from 1.14 to 1.92 TCID_50_-eq/mL. In group 1, two animals remained negative while five animals were positive on one day and one animal on two days during the period 7–9 DPC, with titers ranging from 0.87–2.30 log_10_ TCID_50_-eq/mL.

Comparing the mock-vaccinated group with all vaccinated groups revealed a significant difference (*p* < 0.001) at 4 and 7 DPC. Viral RNA levels in samples collected from group 4 significantly differed from those of groups 1 and 2 on 7 DPC (*p* < 0.001) and 9 (*p* < 0.01) and in leukocytes collected from group 3 and groups 1 and 2 on 7 DPC (*p* < 0.001).

### 3.6. Serology

**Figure 4:**
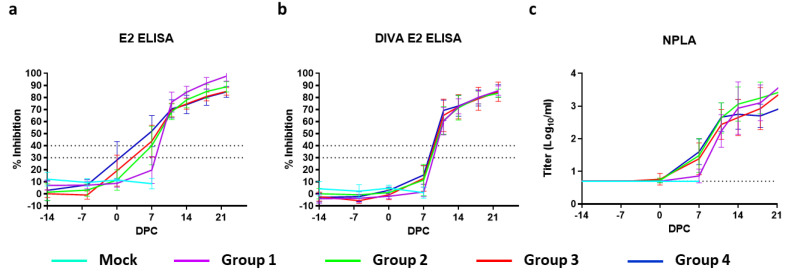
Detection and antibodies by E2 (DIVA) ELISA and NPLA. Results are presented as mean antibody levels (% Inhibition) as determined by the IDEXX E2 ELISA (**a**) and the DIVA E2 ELISA (**b**). Cut-offs (dotted lines) were set at 40% inhibition; ≥30%: doubtful, ≥40%: positive. The cut-off of the NPLA (**c**) was set at 0.7. Error bars represent SD.

Antibody responses were measured using a neutralisation-peroxidase-linked-assay (NPLA), the commercial E2 ELISA (IDEXX), and the modified (DIVA) ELISA [17]. NPLA titers are expressed as the reciprocal of the serum dilution neutralizing 54 TCID_50_/_mL_ of CSFV strain Brescia. Titers of ≥10 are regarded as positive. The mean titers expressed as log_10_ values with SD are depicted in Figure 4a. None of the mock- or C-DIVA-vaccinated piglets contained detectable levels of CSFV-specific neutralizing antibodies before challenge infection, with the exception of one pig from group 3, which had a titer of 15 on 0 DPC. All pigs from group 4 and six out of 8 pigs from group 3 had neutralizing antibodies on 7 DPC. On 11 DPC, all pigs from this group were positive for neutralizing antibodies.

Analysis of the sera with the commercial IDEXX ELISA demonstrated that 7 pigs from group 4, 6 pigs from group 3, 4 pigs from group 2 and 0 pigs from Group 1 seroconverted before challenge, although none of the animals became positive in the 14 days before challenge according to the cut-off of the test (Figure 4a). In contrast, none of the C-DIVA vaccinated pigs developed antibodies that could be detected with the DIVA E2 ELISA until challenge (Figure 4b). Within 11 days after challenge infection, all C-DIVA vaccinated animals became positive in the DIVA ELISA.

### 3.7. Validity and Calculation of the PD50

The PD50 study was found valid because all animals of the mock-vaccinated group developed CSF-specific clinical signs and were euthanized within 7–8 DPC, after reaching a humane end-point. Three PD50s were determined for mortality, clinical signs (including body temperature), and viremia, resulting in PD50 values of ≤0.50, ≤1.88 and ≤2.00, respectively. This suggests that a dose of 0.50, 1.88 and 2.00 log10 TCID50/mL will protect 50% of the animals against mortality, clinical signs and viremia, respectively.

## 4. Discussion

The present work was performed to determine the 50% protective dose (PD50) of the C-DIVA vaccine, when applied as a single, intramuscular vaccination, 14 days before challenge with a highly virulent CSFV strain. Vaccination with 1, 2, 3 or 4 log_10_ TCID_50_ C-DIVA protected pigs from an otherwise lethal dose of the highly virulent Brescia strain, whereas all mock-vaccinated pigs had to be euthanized after reaching a pre-defined humane end-point. Pigs vaccinated with 4 log_10_ TCID_50_ of C-DIVA developed no clinical signs, fever or viremia upon challenge infection, while in the other groups only mild clinical signs (loss of appetite, liveliness and mild fever), and transient, low-level viremia were noted. Surprisingly, only mild clinical signs were observed in challenged pigs that were vaccinated with a very low dose of 1 log_10_ TCID_50_. Viremia levels and duration correlated perfectly with the different vaccine doses administered and the occurrence of viremia was, therefore, added as an additional PD50 parameter.

Despite all pigs were protected from the challenge infection, most pigs were negative for E2 antibodies and neutralizing antibodies at the moment of challenge infection (14 days post vaccination). Antibody levels increased rapidly upon challenge infection, with most pigs scoring positive in the E2 ELISA and NPLA at 7 DPC. Pigs immunized with the lowest dose were all positive for E2 antibodies and neutralizing antibodies by 11 DPC. Importantly, the commercial E2 ELISA does not distinguish between E2-specific antibodies induced by the C-DIVA vaccine virus and those induced by the Brescia challenge virus. Although not within the primary scope of the present study, we analysed the same sera with the modified E2 (DIVA) ELISA as previously described [17]. None of the vaccinated pigs developed antibodies that were detectable in the DIVA ELISA until challenge infection, supporting the applicability of the modified E2 ELISA as a DIVA test [17]. All pigs seroconverted extremely rapidly in this test, with all pigs scoring positive at 11 DPC, suggesting that the DIVA ELISA is extremely sensitive in detecting infected pigs in a vaccinated population.

The most efficacious DIVA vaccine that is currently on the market is the CP7_E2alf vaccine, commercialized by Zoetis as Suvaxyn CSF Marker [13]. In contrast to C-DIVA, which is compatible with a highly sensitive and specific E2-based DIVA ELISA, the CP7_E2alf vaccine has to be accompanied by an E^RNS^ ELISA for DIVA application. There are currently two commercially available E^RNS^ ELISAs: the PrioCHECK CSFV E^RNS^ ELISA (Thermofisher, Waltham, MA, USA) and the “pigtype” CSF Marker ELISA (Qiagen, Hilden, Germany). However, due to suboptimal specificity and sensitivity, both E^RNS^ ELISAs are only recommended to be used at a herd level [14]. As progress is still being made to improve the performance of E^RNS^ ELISAs [26], E2-based ELISAs are still preferred for CSF (DIVA) diagnostics.

## 5. Conclusions

In conclusion, the present work demonstrates that a single, extremely low dose of C-DIVA protects pigs against a highly virulent CSFV strain and that the vaccine can be accompanied by a modified E2 ELISA as a DIVA test. Infection of C-DIVA vaccinated pigs results in a rapid rise in E2 antibodies, detected by the DIVA ELISA, facilitating the detection of infected pigs in a vaccinated population. The strong reduction in challenge virus in oropharyngeal swabs suggests that C-DIVA vaccination also prevents shedding, although this feature remains to be investigated in transmission studies.

## Data Availability

The data presented in this study are available on request from the corresponding author.

## Supplementary Materials

The following are available online at https://www.mdpi.com/article/10.3390/vaccines9050483/s1, Figure S1: Individual rectal temperatures, Table S1: Individual rectal temperatures.
