# Supplementary material for: Assessing the Protective Dose of a Candidate DIVA Vaccine against Classical Swine Fever
Source: Vaccines (Basel). 2021 May 10;9(5):483. doi: 10.3390/vaccines9050483 (PMC8151196; doi:10.3390/vaccines9050483)
Supplement: Supplementary file 1 [file vaccines-09-00483-s001.zip › vaccines-1193054-supplementary.pdf]

Supplementary data

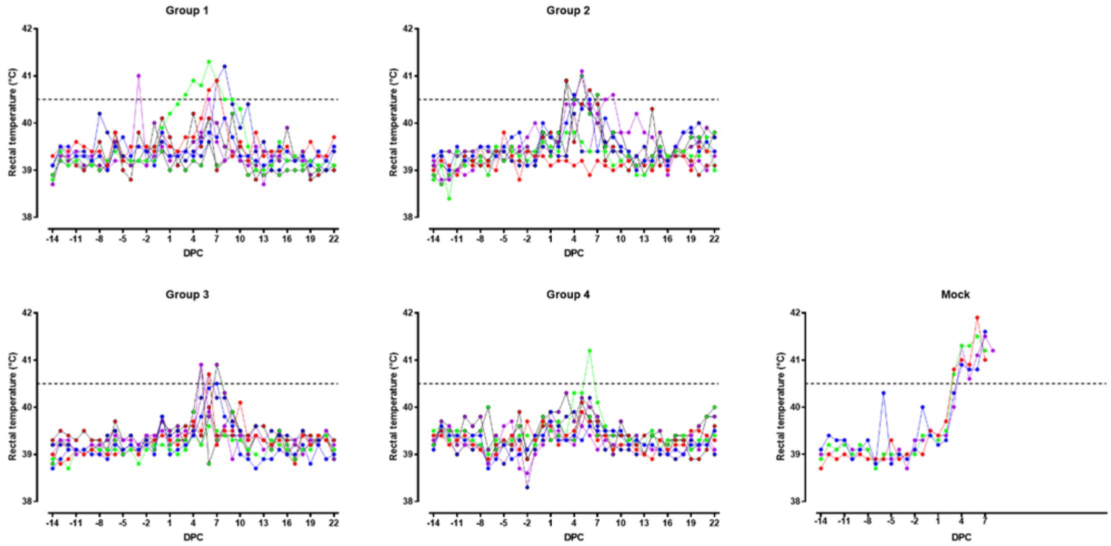

Figure S1. Individual rectal temperatures of piglets vaccinated with 10^1 (Group 1), 10^2 (Group 2), 10^3 (Group 3) or 10^4 (Group 4) $TCID_{50}$ of the C-DIVA vaccine, followed by challenge with wild-type CSFV (Brescia strain) 14 days after vaccination. Temperature above the dashed line is defined as fever. DPC; days post challenge.

DPC	Group 4 (10^4 dose)								Group 3 (10^3 dose)							
-14	39.1	39.4	39.5	39.3	39.3	39.4	39.2	39.2	38.7	39.0	38.9	39.0	39.2	39.3	38.8	39.2
-13	39.6	39.4	39.6	39.4	39.4	39.5	39.6	39.7	38.9	38.8	38.9	39.3	39.2	39.5	39.3	39.3
-12	39.4	39.2	39.3	39.6	39.3	39.3	39.5	39.6	39.1	38.9	38.7	39.3	39.2	39.4	39.2	39.1
-11	39.4	39.3	39.5	39.3	39.0	39.0	39.5	39.2	39.1	39.1	39.1	39.0	39.1	39.3	39.1	39.1
-10	39.3	39.4	39.3	39.2	39.2	39.2	39.5	39.8	39.1	39.0	39.1	39.1	39.0	39.5	39.1	39.1
-9	39.4	39.2	39.3	39.3	39.1	39.2	39.4	39.6	39.0	39.1	39.1	39.2	39.1	39.3	39.2	39.1
-8	39.4	39.1	39.2	39.1	39.2	39.2	39.5	39.8	39.0	39.0	39.1	39.2	39.0	39.3	39.1	39.2
-7	38.7	38.9	39.0	38.8	39.0	38.8	40.0	39.1	38.9	39.0	39.1	39.1	39.4	39.1	39.0	39.3
-6	38.9	39.0	39.3	39.0	39.0	39.2	39.1	39.4	39.2	39.0	39.1	39.4	39.5	39.7	39.3	39.5
-5	39.2	39.2	39.2	39.6	38.8	39.3	39.2	39.2	39.1	39.1	39.1	39.3	39.3	39.3	39.0	39.1
-4	38.9	39.4	39.3	39.7	39.1	39.2	39.4	39.2	39.2	39.2	39.1	39.3	39.4	39.4	39.1	39.2
-3	39.0	39.1	39.4	38.7	39.3	39.9	39.6	39.4	39.1	39.0	38.8	39.2	39.2	39.2	39.1	39.0
-2	39.1	39.7	39.4	38.6	38.3	38.9	39.1	38.9	39.2	39.2	39.1	39.4	39.3	39.4	39.3	39.4
-1	39.4	39.3	39.2	39.0	39.1	39.2	39.4	39.2	39.0	39.3	39.2	39.4	39.2	39.2	39.1	39.3
0	39.4	39.7	39.7	39.2	39.3	39.6	39.8	39.7	39.8	39.4	39.2	39.5	39.8	39.8	39.8	39.7
1	39.7	39.6	39.5	39.5	39.9	39.7	39.3	39.9	39.0	39.3	39.1	39.3	39.4	39.4	39.2	39.5
2	39.3	39.2	39.7	39.5	39.6	39.7	39.4	39.9	39.1	39.2	39.0	39.4	39.5	39.5	39.3	39.6
3	39.3	39.4	39.4	39.3	39.8	39.3	39.3	40.3	39.2	39.4	39.1	39.5	39.4	39.6	39.3	39.1
4	39.4	39.5	40.3	39.2	39.9	39.4	39.3	39.7	39.4	39.7	39.4	39.4	39.5	39.6	39.9	39.5
5	39.3	39.9	40.3	39.6	39.6	40.1	39.9	40.2	39.8	39.5	39.2	40.9	40.2	39.4	40.9	39.2
6	39.6	39.6	41.2	39.4	40.2	39.7	39.8	40.0	40.4	40.7	39.6	39.9	40.7	40.0	38.8	39.8
7	39.5	39.3	40.1	39.1	39.6	39.7	39.4	39.8	40.5	39.2	39.5	39.2	40.2	39.4	39.3	40.9
8	39.5	39.4	39.6	39.3	39.1	39.5	39.4	39.5	39.8	39.5	39.4	39.6	40.2	39.4	39.6	40.3
9	39.5	39.1	39.1	39.2	39.2	39.5	39.4	39.0	39.6	39.5	39.3	38.9	39.7	39.4	39.9	39.9
10	39.5	39.2	39.2	39.2	39.1	39.4	39.4	39.7	39.0	40.1	39.3	39.4	39.5	39.4	39.5	39.4
11	39.2	39.2	39.3	39.2	39.1	39.4	39.2	39.3	38.9	39.3	39.1	39.3	39.3	39.4	39.1	39.0
12	39.3	39.1	39.3	39.3	39.0	39.2	39.2	39.4	38.7	39.4	39.0	39.4	39.6	39.6	39.4	39.4
13	39.2	39.0	39.2	39.2	39.0	39.4	39.0	39.0	38.9	39.3	38.9	39.1	39.3	39.6	39.3	39.3
14	39.2	38.9	39.5	39.1	39.2	39.5	39.4	39.4	38.9	39.2	39.3	39.2	39.5	39.2	39.1	39.2
15	39.1	39.3	39.1	39.1	39.0	39.1	39.3	39.5	39.1	39.3	39.2	39.1	39.4	39.4	39.5	39.5
16	39.0	39.1	39.3	39.1	39.0	39.3	39.2	39.0	39.0	39.0	39.1	39.0	39.4	39.3	39.2	39.0
17	39.1	39.2	39.2	39.1	38.9	39.3	39.4	39.4	39.0	38.8	38.8	39.3	39.3	39.3	38.9	39.2
18	39.0	39.2	39.4	39.0	39.0	39.3	39.4	39.4	39.2	39.4	39.1	39.0	39.5	39.4	39.5	39.1
19	39.2	39.5	39.1	39.4	39.3	38.9	39.1	39.4	38.8	39.2	39.1	39.3	39.3	39.4	39.2	39.2
20	39.2	39.5	39.3	39.1	39.2	38.9	39.2	39.6	39.2	39.4	39.4	39.2	39.3	39.2	39.4	39.3
21	39.2	39.4	39.3	39.4	39.3	39.1	39.8	39.8	38.9	39.3	39.4	39.5	39.4	39.5	39.3	39.4
22	39.5	39.3	39.4	39.1	39.0	39.6	40.0	39.8	39.0	39.0	39.1	39.2	39.1	39.3	39.1	38.9

DPC	Group 2 (10^2 dose)								Group 1 (10^1 dose)								Mock			
-14	39.3	39.0	38.8	39.2	39.0	38.8	38.9	39.1	39.1	39.3	38.8	38.7	39.1	38.7	38.9	39.1	39.1	38.7	38.9	39.0
-13	39.4	39.2	39.1	38.8	39.3	39.1	38.7	39.4	39.5	39.5	39.4	39.3	39.5	39.3	39.2	39.4	39.4	39.0	39.2	39.0
-12	39.0	39.1	38.4	38.8	38.8	39.0	38.9	39.4	39.5	39.4	39.1	39.3	39.3	39.3	39.1	39.2	39.3	38.9	39.1	39.1
-11	39.5	38.9	39.4	39.0	39.0	39.0	39.0	39.3	39.3	39.6	39.2	39.4	39.4	39.1	39.2	39.3	39.3	39.0	39.2	39.3
-10	39.2	39.2	39.1	38.9	39.1	39.3	39.2	39.4	39.3	39.5	39.3	39.3	39.4	39.0	39.0	39.1	38.9	38.9	39.0	39.1
-9	39.3	39.1	39.1	39.0	39.1	39.2	39.2	39.4	39.2	39.4	39.1	39.3	39.2	39.2	39.2	39.1	39.1	39.0	39.2	39.1
-8	39.5	39.5	39.2	39.2	39.2	39.1	39.3	39.5	39.3	39.4	39.3	39.4	40.2	39.6	39.0	39.1	39.3	38.9	39.1	39.1
-7	39.5	39.1	38.9	38.9	39.1	39.2	39.2	39.4	39.0	39.1	39.2	39.0	39.8	39.1	39.1	39.0	38.8	38.9	38.7	38.8
-6	39.3	39.0	39.5	39.3	39.3	39.4	39.5	39.3	39.5	39.8	39.4	39.2	39.5	39.8	39.8	39.6	40.3	38.9	39.0	38.9
-5	39.5	38.8	39.5	39.5	39.4	39.4	39.2	39.2	39.7	39.2	39.2	39.2	39.3	39.0	39.3	39.2	38.8	39.3	39.0	38.9
-4	39.7	39.3	39.4	39.4	39.2	39.2	39.5	39.3	39.3	39.5	39.2	39.2	39.2	38.8	39.2	39.1	39.0	38.9	39.0	39.1
-3	39.8	38.8	39.3	39.5	39.1	39.2	39.3	39.4	39.5	39.5	39.2	41.0	39.2	39.8	39.2	39.2	38.9	39.0	39.0	38.7
-2	39.1	39.4	39.2	39.7	39.1	39.1	39.4	39.5	39.4	39.5	39.2	39.1	39.2	39.4	39.5	39.1	39.1	39.1	39.0	39.3
-1	39.2	39.3	39.4	40.0	39.1	39.1	39.4	39.2	39.1	39.4	39.2	39.3	39.4	39.5	39.2	40.0	40.0	39.0	39.4	39.3
0	40.0	39.3	39.6	39.6	39.5	39.7	39.8	39.8	39.8	39.8	39.9	39.5	39.6	40.1	39.4	39.4	39.4	39.5	39.4	39.5
1	39.7	39.1	39.5	39.5	39.4	39.8	39.3	39.7	39.3	39.5	40.2	39.2	39.5	39.7	39.0	39.3	39.2	39.4	39.3	39.4
2	39.5	39.2	39.4	39.5	39.3	39.5	39.8	39.6	39.3	39.4	40.4	39.4	39.4	39.1	39.3	39.4	39.3	39.7	39.5	39.4
3	40.0	39.2	39.8	40.4	39.3	40.9	39.3	40.9	39.4	39.7	40.6	39.4	39.2	39.4	39.0	39.4	40.3	40.8	40.7	40.0
4	40.6	39.1	39.8	40.4	40.2	39.6	40.5	40.5	39.3	39.7	40.9	39.3	39.4	40.2	39.2	39.6	40.9	41.0	41.3	41.3
5	40.3	39.2	39.6	41.1	39.4	40.4	41.0	40.4	39.6	40.1	40.8	39.8	39.3	39.5	39.1	39.7	40.8	40.9	41.3	40.6
6	40.5	38.9	39.4	40.4	39.4	40.7	40.2	40.3	39.8	40.7	41.3	40.5	39.6	40.1	39.6	40.1	40.8	41.9	41.5	41.1
7	39.4	39.2	39.4	40.2	40.2	40.4	40.6	40.0	39.7	40.9	40.9	39.6	40.9	39.0	39.1	40.0	41.6	41.0	41.2	41.5
8	40.1	39.1	39.4	40.5	39.6	39.2	39.5	39.5	40.1	40.1	40.5	39.2	41.2	39.2	39.2	39.5				41.2
9	39.7	39.0	39.1	40.6	39.4	39.5	39.8	39.8	39.7	39.3	40.5	39.4	40.4	39.3	40.2	39.4				
10	39.5	39.1	39.2	39.8	39.4	39.2	39.8	39.4	39.3	39.6	40.3	39.2	39.9	39.5	39.3	39.2				
11	39.4	39.2	39.4	39.8	39.2	39.0	39.3	39.2	39.3	39.2	39.5	39.1	40.4	39.3	38.9	39.2				
12	39.0	39.0	38.9	40.2	39.3	39.3	39.1	39.3	39.2	39.8	39.0	39.5	39.4	38.8	39.0	39.1				
13	39.2	39.2	38.9	39.8	39.5	39.1	39.2	38.9	39.1	39.3	39.0	38.7	39.6	39.1	38.9	39.4				
14	39.2	39.0	39.1	39.7	39.0	40.3	39.3	39.2	39.2	39.4	39.1	39.3	39.0	39.1	39.0	39.4				
15	39.4	39.4	39.4	39.4	39.3	39.1	39.5	39.8	39.5	39.4	39.6	39.3	39.0	39.3	38.9	39.2				
16	39.1	39.0	39.3	38.9	39.2	39.3	39.3	39.2	39.4	39.5	39.2	39.4	39.2	39.2	39.0	39.9				
17	39.5	39.5	39.4	39.4	39.4	39.3	39.3	39.5	39.2	39.2	39.3	39.3	39.2	39.2	39.0	39.2				
18	39.8	39.3	39.4	39.4	39.8	39.3	39.4	39.4	39.3	39.3	39.1	39.2	39.2	39.3	39.0	39.4				
19	39.9	39.0	39.3	39.1	39.8	39.2	39.7	39.5	39.1	39.6	39.2	39.2	39.2	38.8	39.1	38.9				
20	39.4	39.5	39.3	39.2	40.0	38.9	39.7	39.6	39.4	39.3	39.1	39.3	39.1	38.9	39.0	39.0				
21	39.6	39.4	39.7	39.0	39.7	39.1	39.4	39.9	39.2	39.3	39.2	39.2	39.0	39.0	39.0	39.2				
22	39.4	39.4	39.0	39.3	39.7	39.1	39.8	39.7	39.5	39.7	39.1	39.5	39.4	39.0	39.1	39.1				

Table S1. Individual rectal temperatures of piglets vaccinated with 10^1 (Group 1), 10^2 (Group 2), 10^3 (Group 3) or 10^4 (Group 4) TCID50 of the C-DIVA vaccine, followed by challenge with wild-type CSFV (Brescia strain) 14 days after vaccination. One group of 4 piglets was mock-vaccinated. Temperatures between 40°C and 40.5°C are highlighted in red; Temperatures >40.5°C are highlighted in green.
